# Supplementary material for: Smooth Interpolating Curves with Local Control and Monotone Alternating Curvature
Source: Comput Graph Forum. 2022 Oct 6;41(5):25–38. doi: 10.1111/cgf.14600 (PMC9827861; doi:10.1111/cgf.14600)
Supplement: Supplementary file 1 — Supplement Material [file CGF-41-25-s001.zip › Local-Smooth-Interpolating-MonoCurvature/extern/clothoids/submodules/Utils/ThirdParties/fmt/fmt-8.0.1.zip!/fmt-8.0.1/doc/basic-bootstrap/layout.html]

{#
basic/layout.html
~~~~~~~~~~~~~~~~~
Master layout template for Sphinx themes.
:copyright: Copyright 2007-2015 by the Sphinx team, see AUTHORS.
:license: BSD, see LICENSE for details.
#}
{%- block doctype -%}
{%- endblock %}
{%- set reldelim1 = reldelim1 is not defined and ' »' or reldelim1 %}
{%- set reldelim2 = reldelim2 is not defined and ' |' or reldelim2 %}
{%- set render\_sidebar = (not embedded) and (not theme\_nosidebar|tobool) and
(sidebars != []) %}
{%- set url\_root = pathto('', 1) %}
{# XXX necessary? #}
{%- if url\_root == '#' %}{% set url\_root = '' %}{% endif %}
{%- if not embedded and docstitle %}
{%- set titlesuffix = " — "|safe + docstitle|e %}
{%- else %}
{%- set titlesuffix = "" %}
{%- endif %}
{%- macro relbar() %}

### {{ \_('Navigation') }}

{%- for rellink in rellinks %}- {{ rellink[3] }}
  {%- if not loop.first %}{{ reldelim2 }}{% endif %}
{%- endfor %}
{%- block rootrellink %}- {{ shorttitle|e }}{{ reldelim1 }}
{%- endblock %}
{%- for parent in parents %}- {{ parent.title }}{{ reldelim1 }}
{%- endfor %}
{%- block relbaritems %} {% endblock %}

{%- endmacro %}
{%- macro sidebar() %}
{%- if render\_sidebar %}

{%- block sidebarlogo %}
{%- if logo %}

{%- endif %}
{%- endblock %}
{%- if sidebars != None %}
{#- new style sidebar: explicitly include/exclude templates #}
{%- for sidebartemplate in sidebars %}
{%- include sidebartemplate %}
{%- endfor %}
{%- else %}
{#- old style sidebars: using blocks -- should be deprecated #}
{%- block sidebartoc %}
{%- include "localtoc.html" %}
{%- endblock %}
{%- block sidebarrel %}
{%- include "relations.html" %}
{%- endblock %}
{%- block sidebarsourcelink %}
{%- include "sourcelink.html" %}
{%- endblock %}
{%- if customsidebar %}
{%- include customsidebar %}
{%- endif %}
{%- block sidebarsearch %}
{%- include "searchbox.html" %}
{%- endblock %}
{%- endif %}

{%- endif %}
{%- endmacro %}
{%- macro script() %}
{%- for scriptfile in script\_files %}
{%- endfor %}
{%- endmacro %}
{%- macro css() %}

{%- for cssfile in css\_files %}
{%- endfor %}
{%- endmacro %}

{# The above 3 meta tags \*must\* come first in the head; any other head content
must come \*after\* these tags. #}
{{ metatags }}
{%- block htmltitle %}
{{ title|striptags|e }}{{ titlesuffix }}
{%- endblock %}
{{ css() }}
{%- if not embedded %}
{{ script() }}
{%- if use\_opensearch %}
{%- endif %}
{%- if favicon %}
{%- endif %}
{%- endif %}
{%- block linktags %}
{%- if hasdoc('about') %}
{%- endif %}
{%- if hasdoc('genindex') %}
{%- endif %}
{%- if hasdoc('search') %}
{%- endif %}
{%- if hasdoc('copyright') %}
{%- endif %}
{%- if parents %}
{%- endif %}
{%- if next %}
{%- endif %}
{%- if prev %}
{%- endif %}
{%- endblock %}
{%- block extrahead %} {% endblock %}

{%- block header %}{% endblock %}
{%- block relbar1 %}{{ relbar() }}{% endblock %}
{%- block content %}
{%- block sidebar1 %} {# possible location for sidebar #} {% endblock %}

{%- block document %}

{%- if render\_sidebar %}

{%- endif %}

{% block body %} {% endblock %}

{%- if render\_sidebar %}

{%- endif %}

{%- endblock %}
{%- block sidebar2 %}{{ sidebar() }}{% endblock %}

{%- endblock %}
{%- block relbar2 %}{{ relbar() }}{% endblock %}
{%- block footer %}

{%- if show\_copyright %}
{%- if hasdoc('copyright') %}
{% trans path=pathto('copyright'), copyright=copyright|e %}© Copyright {{ copyright }}.{% endtrans %}
{%- else %}
{% trans copyright=copyright|e %}© Copyright {{ copyright }}.{% endtrans %}
{%- endif %}
{%- endif %}
{%- if last\_updated %}
{% trans last\_updated=last\_updated|e %}Last updated on {{ last\_updated }}.{% endtrans %}
{%- endif %}
{%- if show\_sphinx %}
{% trans sphinx\_version=sphinx\_version|e %}Created using Sphinx {{ sphinx\_version }}.{% endtrans %}
{%- endif %}

{%- endblock %}
